# Primary Epithelioid Angiosarcoma of the Right Atrium Presenting With Symptomatic Bradycardia: A Case Report

**DOI:** 10.7759/cureus.108865

**Published:** 2026-05-14

**Authors:** Omar Sadaqah, Bahy Abofrekha, Ahmed Zayed, Abdelrahman Abouelnas, Ekrem Yetiskul, Gil Garber, Chadi Salmane

**Affiliations:** 1 Internal Medicine, Staten Island University Hospital, New York City, USA; 2 Pathology, Staten Island University Hospital, New York City, USA; 3 Interventional Cardiology, Northwell Health, New York, USA

**Keywords:** cardio-oncology, epithelioid angiosarcoma, multimodal cardiac imaging, primary cardiac angiosarcoma, right atrial cardiac mass

## Abstract

Primary cardiac angiosarcoma is a rare and aggressive malignancy that mainly affects the right atrium of the heart. In this report, we present a rare variant known as cardiac epithelioid angiosarcoma. Due to its rarity, the epidemiology and pathogenesis of this disease are not well understood.

We present the case of a 44-year-old woman who presented with palpitations, chest pain, and symptomatic bradycardia. Multimodal imaging identified a large, sessile right atrial mass. The location of the mass near the sinoatrial node region likely contributed to the patient’s bradycardia. The patient underwent surgical resection with bovine pericardial patch reconstruction. Histopathology favored epithelioid angiosarcoma, and the margins were unclear due to specimen fragmentation. The postoperative course was complicated by junctional rhythm bradyarrhythmia requiring pacing. Comprehensive staging, including brain MRI and CT of the chest, abdomen, and pelvis, ruled out distant metastasis. The patient was deemed not to be a candidate for radiation therapy due to the tumor’s location in the heart. Although adjuvant AIM chemotherapy (doxorubicin, ifosfamide, mesna) was recommended by oncology, the patient opted for observation, given the limited expected benefit of chemotherapy.

This case highlights the complexity of managing primary cardiac angiosarcoma, particularly when standard therapeutic options are limited, and underscores the need for individualized, multidisciplinary care.

## Introduction

Primary cardiac neoplasms are exceptionally rare, with an incidence of 0.0001%-0.030% in autopsy series [1]. While the majority of these tumors are benign myxomas, angiosarcomas represent the most common primary cardiac malignancy [1]. Epithelioid angiosarcoma represents a rare histologic variant of primary cardiac angiosarcoma. Cardiac angiosarcomas are characterized by rapid, infiltrative growth and a predilection for the right atrium, often leading to early metastasis and obstruction of right-sided inflow [2]. The prognosis is generally poor, with a high rate of local recurrence even after surgical excision [2].

Clinical manifestations are often nonspecific, as cardiac tumors can mimic a wide range of cardiovascular conditions. Patients may present with symptoms such as chest pain, dyspnea, palpitations, or arrhythmias, frequently leading to delayed diagnosis [3]. Early detection remains challenging, as these tumors may not be readily identifiable on initial imaging and are often diagnosed at an advanced stage.

We report a case of primary epithelioid angiosarcoma in a young woman, emphasizing its aggressive nature, propensity for delayed diagnosis, and the pivotal role of multimodal imaging in defining tumor extent and guiding surgical management. Given the rarity of this entity and the lack of consensus on optimal adjuvant therapy, this case contributes to the limited body of literature and highlights the need for an individualized, multidisciplinary approach to care.

## Case presentation

History and physical examination

A 44-year-old woman with no significant past medical history presented to the Emergency Department with a several-week history of palpitations, shortness of breath, and intermittent retrosternal burning chest pain radiating to the neck and jaw. Notably, she experienced episodes of symptomatic bradycardia, with heart rates dropping to 30-40 bpm, significantly lower than her reported baseline. Physical examination on admission revealed a resting heart rate of 50 bpm, but she was hemodynamically stable without signs of overt heart failure.

Diagnostic imaging

**Figure 1:**
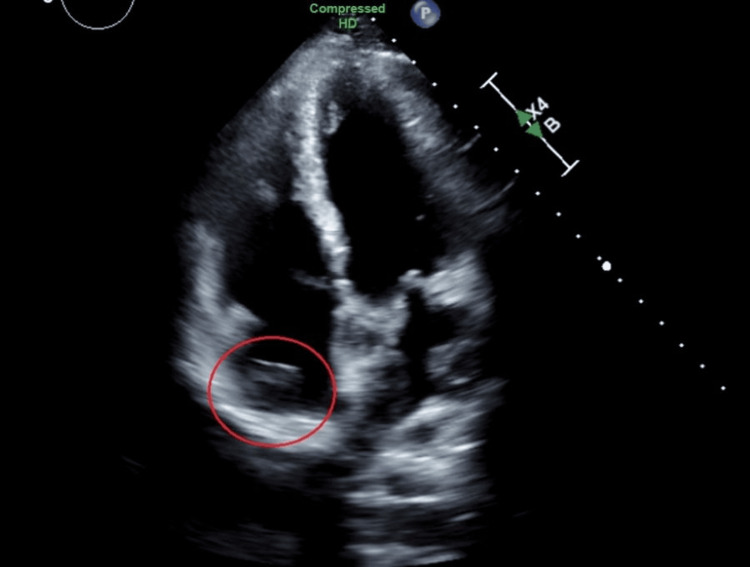
Transthoracic echocardiogram (TTE), apical four-chamber view, demonstrating an echogenic right atrial mass (circled).

**Figure 2:**
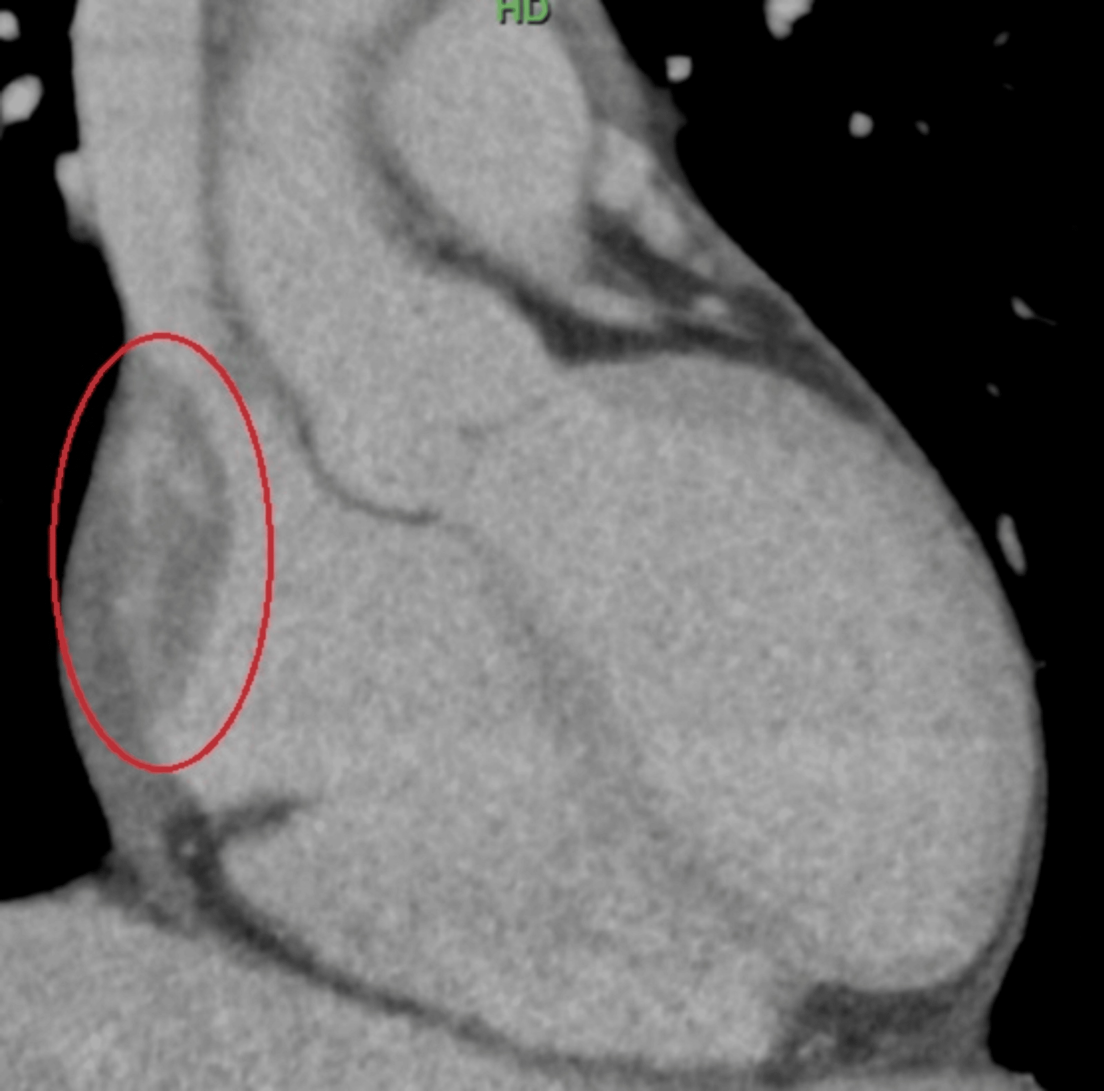
Preoperative cardiac computed tomography (CT). CT chest showing an irregular filling defect (red oval) in the infero-posterior aspect of the right atrium adjacent to the inferior vena cava, extending superiorly to the junction of the superior vena cava, measuring up to 4.5 cm in length (from the superior vena cava (SVC) junction to the inferior right atrium) and 1.5 cm in diameter.

**Figure 3:**
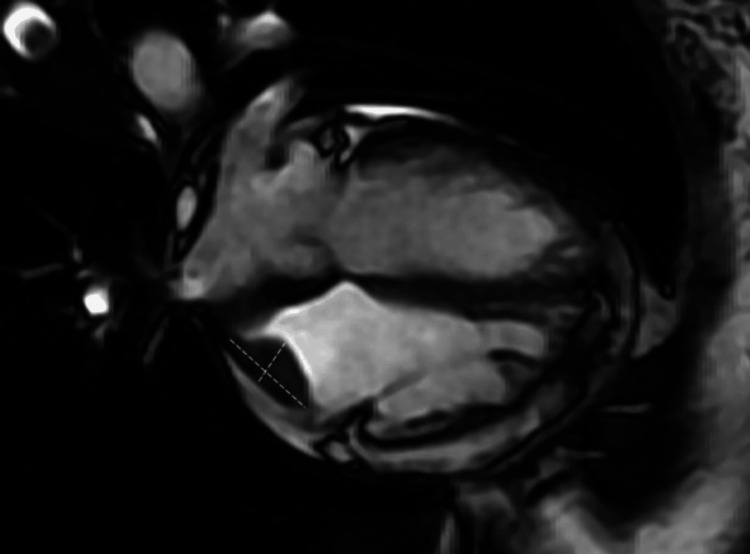
Preoperative cardiac magnetic resonance imaging (MRI). Horizontal long-axis (four-chamber) view; dashed calipers denote the measured dimension (3.0 cm × 1.5 cm) of the mass in the right atrium adherent to the inferoposterior wall.

The patient underwent a comprehensive imaging evaluation. Initial transthoracic echocardiography (TTE) revealed a 1.0 × 1.2 cm echogenic mass in the right atrium (Fig. 1). A cardiac computed tomography (CT) angiogram subsequently demonstrated an ill-defined, irregular filling defect in the inferoposterior right atrium (Fig. 2). Cardiac magnetic resonance imaging (MRI) provided superior tissue characterization, revealing a larger 3.0 cm × 1.5 cm well-circumscribed, sessile mass (Fig. 3). The mass was adherent to the inferoposterior wall of the right atrium and extended superiorly toward the superior vena cava (SVC) junction.

Surgical management

**Figure 4:**
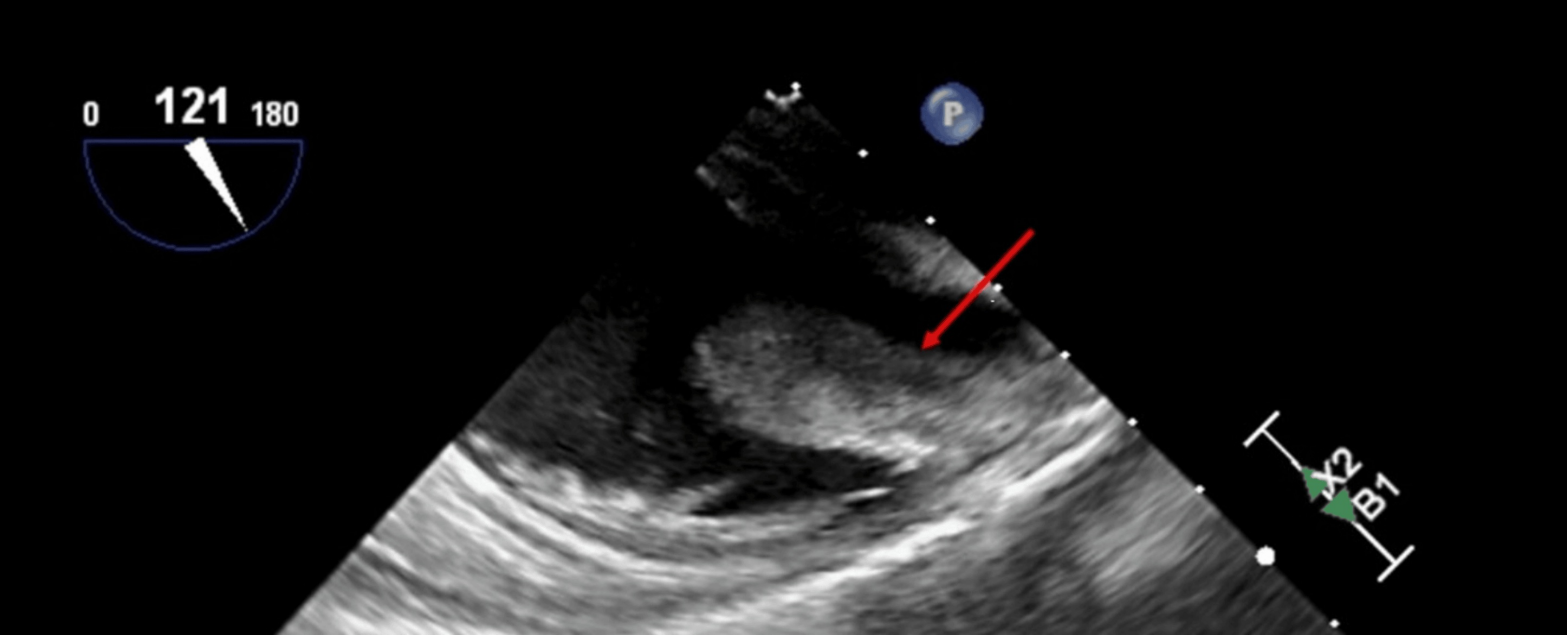
Intraoperative transesophageal echocardiogram (TEE). Mid-esophageal bicaval view demonstrating a 3.2 cm × 1.13 cm echogenic mass (red arrow) anchored at the junction of the superior vena cava (SVC) and right atrium (RA).

The patient was taken to the operating room. An intraoperative transesophageal echocardiogram (TEE) performed prior to bypass visualized a 3.2 × 1.13 cm mass anchored at the SVC-right atrial junction (Fig. 4). This view confirmed the tumor’s proximity to the SVC, guiding the cannulation strategy.

The patient was placed on cardiopulmonary bypass (CPB) for 98 minutes. The right atrium was opened, and the mass was excised. Due to the infiltrative nature of the tumor, the right atrial wall was reconstructed using a bovine pericardial patch. The patient was successfully weaned from bypass with a postoperative left ventricular ejection fraction (LVEF) of 60%.

Pathology

**Figure 5:**
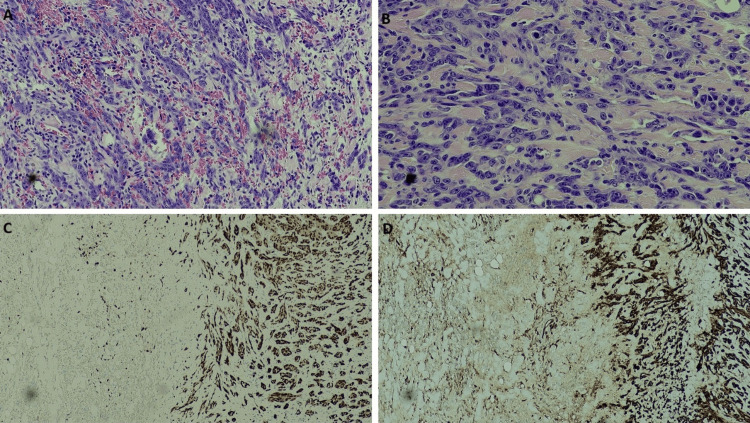
Histopathology images. (A) Hematoxylin and eosin (H&E) staining demonstrating malignant endothelial cells with epithelioid morphology, nuclear pleomorphism, and areas of small vessel formation containing blood. (B) High-power view demonstrating increased mitotic activity and prominent nucleoli. (C) Immunohistochemical staining positive for ERG.  (D) Immunohistochemical staining positive for CD31.

Histopathology showed a moderately cellular malignant vascular neoplasm involving cardiac muscle. The specimen was received in multiple fragments, with the largest fragment measuring 3.8 × 1.9 × 1.5 cm. The extent of vasoformation and immunostaining favored epithelioid angiosarcoma over malignant epithelioid hemangioendothelioma (Fig. 5). The tumor exhibited high-risk features, including high mitotic activity (14 mitoses per 10 high-power fields (HPFs)), necrosis involving 2% of the sample, and unclear margins due to specimen fragmentation. Immunostains were positive for ERG, CD31, and CD34, with scattered AE1/3 expression, and negative for HHV-8, c-Myc, desmin, SMA, and ALK-D5F3.

Postoperative course and staging

The postoperative course was complicated by junctional rhythm bradyarrhythmia with heart rates in the 50s requiring temporary pacing, which subsequently recovered to sinus bradycardia without the need for permanent pacemaker placement. A postoperative surveillance TTE showed a preserved LVEF of 50-55% but identified a 0.8 × 0.8 cm echodensity near the patch site.

Comprehensive staging was performed to rule out distant metastasis. Brain MRI demonstrated no evidence of intracranial metastasis. Follow-up CT chest demonstrated no evidence of metastatic disease in the chest. CT imaging of the abdomen and pelvis revealed no suspicious findings, ruling out visceral metastasis. 

## Discussion

Primary cardiac angiosarcoma is an exceptionally rare and aggressive malignancy that often presents with nonspecific symptoms, leading to delayed diagnosis and advanced disease at detection. Metastatic cardiac tumors are significantly more common than primary tumors, occurring approximately 20-40 times more frequently, further highlighting the rarity of primary cardiac malignancies. These tumors most commonly present in the third to fifth decades of life and demonstrate a male predominance, with reported male-to-female ratios of approximately 2-3:1 [4].

This case highlights the complementary roles of cardiac imaging. While TTE is the standard screening tool, it significantly underestimated tumor size (1.2 cm vs. 3.2 cm on TEE). This case also underscores the limitations of initial TTE assessment and highlights the incremental diagnostic value of TEE and cardiac MRI in accurately characterizing cardiac masses. Cardiac MRI served as the gold standard for tissue characterization, distinguishing tumor from thrombus via gadolinium enhancement. Intraoperative TEE was indispensable for defining the tumor's relationship to the SVC.

Histopathologic evaluation remains the gold standard for diagnosis. Microscopic examination demonstrates malignant endothelial cells forming irregular vascular channels, along with areas of anaplastic cellular proliferation [1]. CD31 is a sensitive and specific marker of vascular tumors, whether benign or malignant. CD34 is also a specific endothelial marker, although it is generally less sensitive than CD31 [2,5,6].

The diagnosis of epithelioid angiosarcoma carries a poor prognosis due to high rates of local recurrence and metastasis [2]. Patients who undergo complete surgical resection demonstrate the best survival outcomes [7]. In this case, the unclear margins and high mitotic rate placed the patient in a high-risk category. Although randomized data are limited for primary cardiac tumors, data from soft tissue sarcoma trials support the use of adjuvant chemotherapy. The AIM protocol (doxorubicin, ifosfamide, mesna) has been associated with improved recurrence-free survival and a modest overall survival benefit in soft tissue sarcomas. Meta-analyses of randomized controlled trials have demonstrated an absolute survival benefit of up to approximately 11% with combination regimens including doxorubicin and ifosfamide [8]. More recent analyses suggest that this benefit is not uniform and appears to be primarily confined to selected high-risk patients with adverse prognostic features [9]. These findings underscore the importance of individualized treatment decisions when considering adjuvant chemotherapy.

The delivery of radiotherapy in cardiac tumors is technically challenging due to cardiac and respiratory motion, which makes precise targeting difficult without affecting adjacent healthy tissue. This limitation restricts both the total dose and dose per fraction. Nevertheless, radiotherapy has been shown to improve progression-free survival [10]. Radiation therapy was not considered feasible in this patient due to the tumor’s cardiac location and the high risk of complications.

## Conclusions

Primary cardiac angiosarcoma is a rare and aggressive malignancy that often presents with nonspecific cardiac symptoms, leading to delayed diagnosis. This case highlights the importance of multimodal imaging, particularly cardiac MRI and intraoperative TEE, in accurately defining tumor extent and guiding surgical planning. Given the absence of standardized adjuvant treatment strategies, management requires individualized multidisciplinary decision-making.
